# Clinical features and treatment of 7 Chinese TAFRO syndromes from 96 de novo Castleman diseases: a 10-year retrospective study

**DOI:** 10.1007/s00432-019-03120-w

**Published:** 2020-01-14

**Authors:** Yi Zhang, Shan-Shan Suo, Han-Jin Yang, Xin-Ping Zhou, Liang-Shun You, Wen-Juan Yu, Zhao-Ming Wang, Jie Jin

**Affiliations:** 1 Department of Hematology, The First Affiliated Hospital, College of Medicine, Zhejiang University, 79 Qingchun Road, Hangzhou, 310003 P. R. China; 2 Zhejiang Province Key Laboratory of Hematology Oncology, Diagnosis and Treatment, Hangzhou, China; 3 Institute of Hematology, Zhejiang University, Hangzhou, Zhejiang China; 4 Department of Pathology, The First Affiliated Hospital, College of Medicine, Zhejiang University, 79 Qingchun Road, Hangzhou, 310003 P. R. China

**Keywords:** Giant lymph node hyperplasia, Castleman disease, TAFRO syndrome, Diagnosis, Therapeutics

## Abstract

**Background:**

Castleman disease (CD) is a rare polyclonal lymphoproliferative disorder with unknown etiology. TAFRO syndrome is now regarded as a specific subtype of CD, and is still a huge challenge for clinicians.

**Methods:**

To clarify the clinical features and management of TAFRO syndrome in China, we retrospectively analyzed 96 patients with HIV-negative CD (52 with unicentric CD and 44 with multicentric CD), who were diagnosed and treated at our center between 2008 and 2017. Specially, we systematically reviewed the 7 TAFRO syndrome cases based on the 2015 criteria proposed by Masaki.

**Results:**

Among the 7 cases, there were 3 men and 4 women, and the median age was 53 years. The main symptoms included thrombocytopenia (7/7), anasarca (7/7), fever (4/7), renal dysfunction (7/7), and organomegaly (6/7). One patient was treated with corticosteroid monotherapy, one received RD (Rituximab, dexamethasone), and 5 received CHOP/COP like chemotherapy as first-line treatment, 2 of the 5 combined with Rituximab. Four patients needed hemodialysis or CRRT because of progressive renal failure. The outcome for TAFRO syndrome was significantly worse compared to other types of CD. Although 3 patients improved after early treatment, 4 patients died due to disease progression, and only one patient achieved complete resolution of all the symptoms after changing to lenalidomide based regimen.

**Conclusions:**

This study reveals that TAFRO syndrome is more severe and has more systemic symptoms than other iMCD, most cases need active treatment, and their prognoses are poor. Lenalidomide based regimen may be as a promising new therapy for TAFRO syndrome.

## Introduction

Castleman disease (CD), also known as giant lymph node hyperplasia, is a rare polyclonal lymphoproliferative disorder that was first described by Dr. Benjanmin Castleman in 1954 (Castleman et al. 1956; Castleman and Towne 1954). According to the lesions involved, CD can be classified as unicentric CD (UCD) and multicentric CD (MCD). UCD only involves a single lymph node region, with minimal symptoms and is often treated with localized surgical removal (Wong 2018). In contrast, MCD manifests with widespread lymphadenopathy, has constitutional symptoms, and systemic therapy is always required. Histologically, CD can be divided into hyaline vascular (HV), plasma cell (PC), and mixed cellular (Mix) subtypes. The HV subtype is common in UCD, and the PC subtype in MCD (Astle et al. 2018; Dong et al. 2015). Compared with UCD, MCD has a significantly inferior prognosis (Zhang et al. 2018). In 2014, Fajgenbaum et al. proposed the concept of idiopathic multicentric Castleman disease (iMCD), which defines as MCD patients with negative human herpesvirus (HHV-8) and human immunodeficiency virus (HIV), which accounts for about 50% of MCD (Fajgenbaum et al. 2014, 2017). It is currently believed that iMCD can be classified into TAFRO syndrome and idiopathic multicentric Castleman disease-not otherwise specified (iMCD-NOS) based on clinical characteristics, pathological features, and laboratory results (Carbone and Pantanowitz 2016; Igawa and Sato 2018).

TAFRO (or Castleman-Kojima) syndrome has been gradually recognized in recent years. It is a systemic inflammatory disease characterized by thrombocytopenia (T), anasarca (A), myelofibrosis/fever (F), renal dysfunction/reticulin fibrosis (R), and organomegaly (O). Kojima et al. (2008) described the clinical and pathological findings of the idiopathic plasmacytic lymphadenopathy (IPL) and non-IPL. TAFRO syndrome was first reported by Takai et al. (2010). Currently, we may regard TAFRO syndrome as a type of non-IPL-type MCD. In 2012, the Fukushima (6 June 2012) and Nagoya (22 September 2012) meetings in Japan defined TAFRO syndrome as a systemic inflammatory disease characterized by a series of clinical symptoms, and also discussed the diagnosis and treatment options for this disease (Kawabata et al. 2013). The optimal treatment for TAFRO syndrome is unknown, some patients are treated with corticosteroids and/or immunosuppressive agents, and some are treated with chemotherapy or combined with novel drugs such as monoclonal antibody of CD20 antigen (Rituximab), anti-human interleukin-6 (IL-6) receptor antibody (Tocilizumab), or immunomodulator (Thalidomide, Lenalidomide) (Fujiwara et al. 2016; Igawa and Sato 2018). Up to now, most of the understanding for TAFRO syndrome comes from rare case reports and smaller case series, and few of the cases were Chinese. Thus, the purpose of this study is to clarify the strategies for diagnosing and treating of Chinese TAFRO syndrome. Here, we described the clinical features, actual treatments, as well as the outcomes of 7 Chinese patients diagnosed with TAFRO syndrome among 96 HIV- negative CD in our center.

## Materials and methods

### Patients

**Fig. 1:**
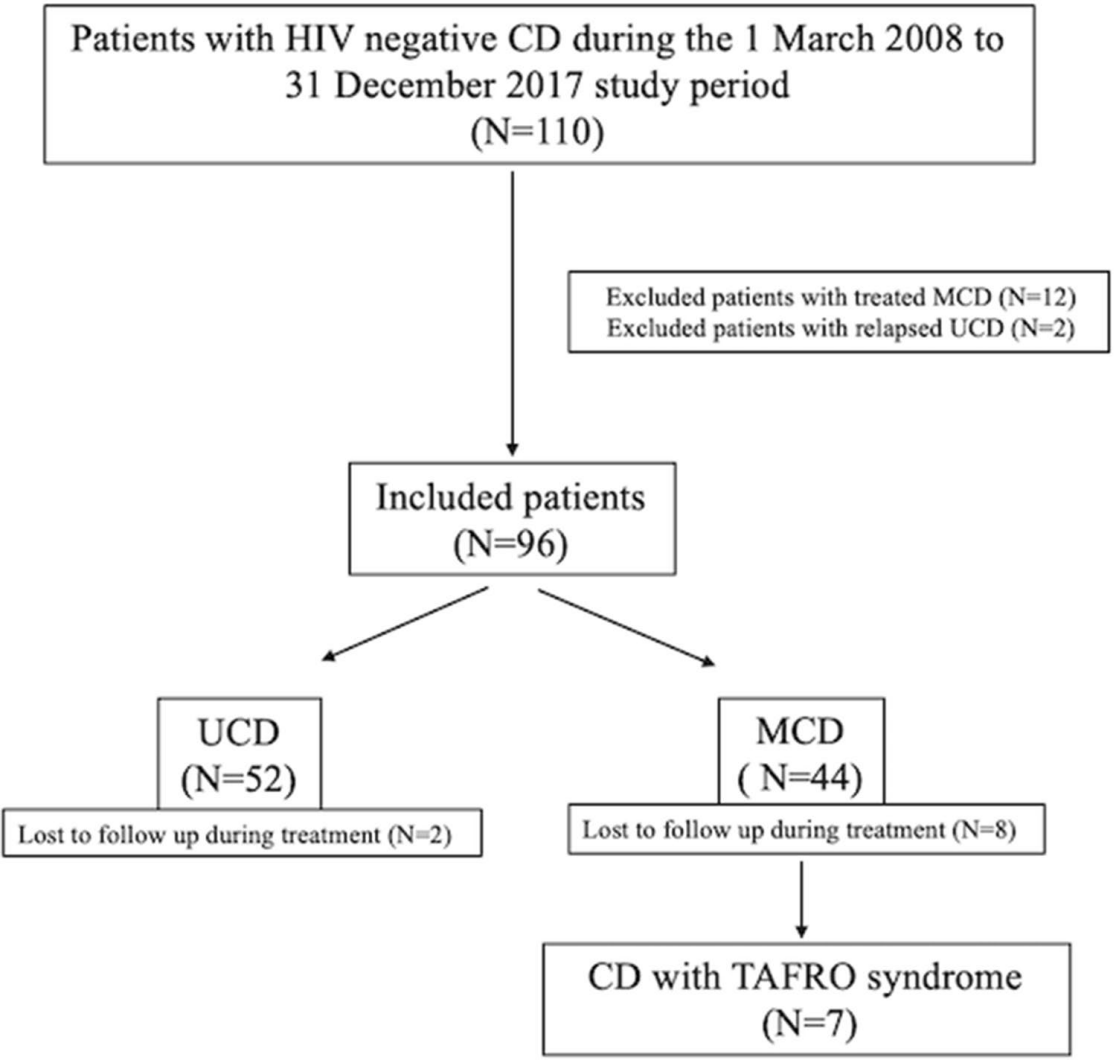
Flow diagram of patient recruitment

We identified 110 HIV-negative CD cases with the confirmed clinical and pathological diagnoses who were admitted to our hospital between March 2008 and December 2017. After review of the medical records, 12 MCD patients whose primary treatments were not in our center, and 2 UCD patients admitted because of disease relapses were excluded. The remaining 96 de novo CD patients were enrolled, Fig. 1. All data were collected through telephone and medical records. Latest follow-up was June 2019. Written informed consent was obtained from all patients in accordance with the Declaration of Helsinki, and the study was approved by the ethics committee of the First Affiliated Hospital of Zhejiang University.

### Criteria

The diagnosis and severity classification of TAFRO syndrome are based on the criteria proposed by Masaki. Thrombocytopenia was defined as platelet count < 100 × 10^9^/L. Pleural effusion and/or ascites were diagnosed by computed tomography (CT) scans or B ultrasonography. The diagnosis of myelofibrosis depended on bone marrow biopsy. Fever was defined as a temperature > 37.5 °C, and renal dysfunction as elevated serum creatinine levels above the reference range or GFR < 60 mL/min/1.73 m^2^. Spleen and liver size was evaluated by CT scans. Survival time was defined as the period from diagnosis to either death or the last follow-up.

### Statistical analysis

All statistical analyses were performed using SPSS Version 25. Continuous variables were described as median (range) analyzed by Mann–Whitney *U* test, and categorical variables were described as frequency (percentage) compared by Pearson *χ*^2^ test. The Kaplan–Meier method was used for survival analysis, and the log-rank test was applied to compare the survival rate between groups. A two-tailed* P* < 0.05 was considered significant.

## Results

### Patient characteristics

**Fig. 2:**
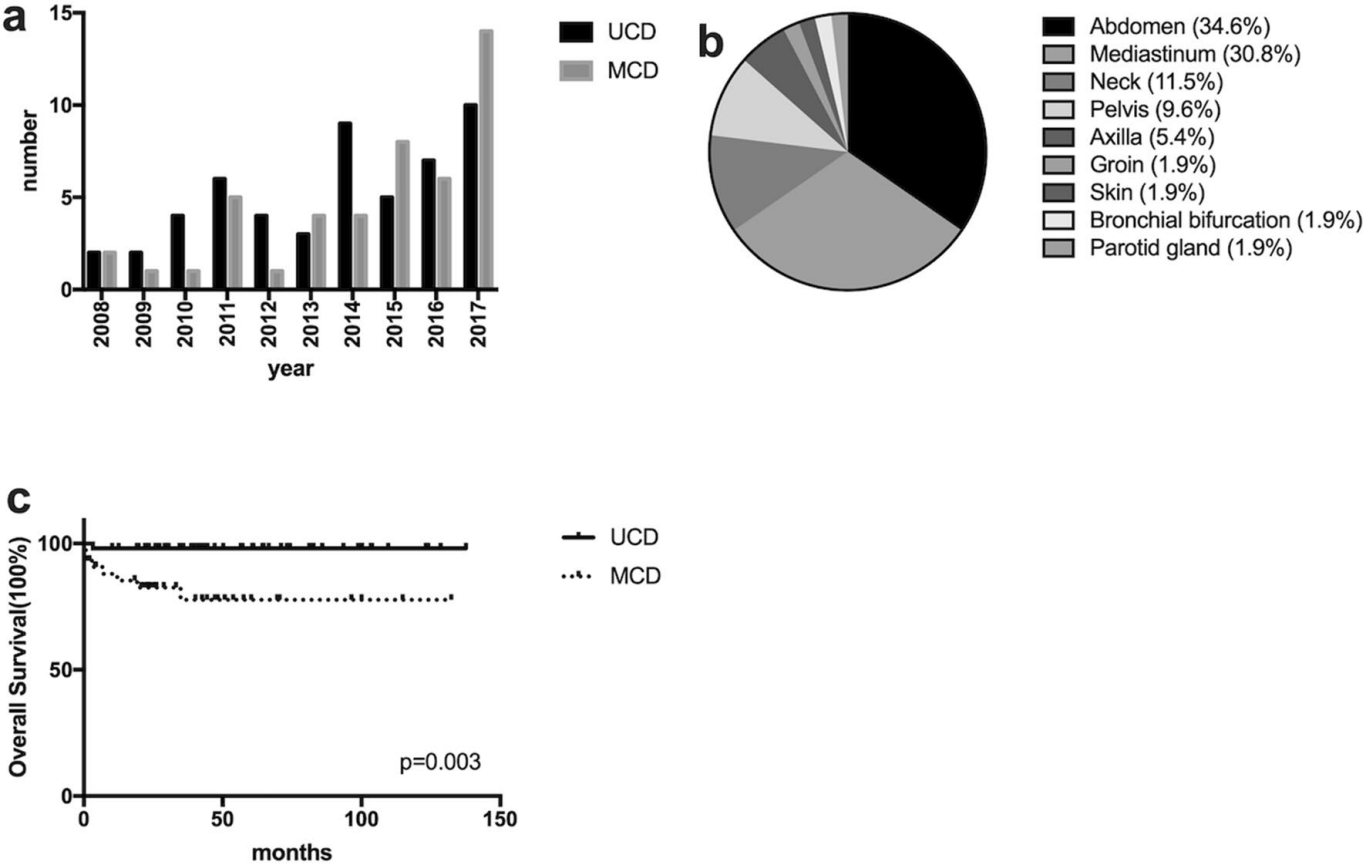
The comparison of characteristics and outcomes between unicentric and multicentric Castleman disease. **a** The annual onset of unicentric (UCD) and multicentric (MCD) Castleman disease; **b** the distribution of lymphadenopathy among patients with UCD; **c** overall survival of patients with UCD and MCD

The number of CD diagnosed and treated in our center was increasing year by year, Fig. 2a. In total we analyzed 96 patients, the median age was 46.5 (range 14–77) years old and 49 (51.0%) were male. Within this cohort, 52 (54.2%) cases were classified with UCD, while the remaining 44 (45.8%) cases were MCD. Sites of lymph node involvement in UCD included abdomen (34.6%), mediastinum (30.8%), neck (11.5%), pelvis (9.6%), axilla (5.4%), groin (1.9%) etc. Sites such as skin, bronchial bifurcation and parotid gland were also involved in our cases, Fig. 2b. In all CD, there were HV for 55 (57.3%), PC for 31 (32.3%), and Mix for 10 (10.4%). The patient’s first visit departments included oncology surgery, thoracic surgery, hepatobiliary surgery, hematology, infection, nephrology, etc. The main manifestations included painless swelling of lymph nodes and in some case spleen, liver or other organomegaly, followed by B symptoms (fever, weight loss and fatigue), and in several cases the initial clinical symptoms were shortness of breath caused by pleural effusion or bloating caused by ascites. Other patients were diagnosed accidentally by physical exam. Among the 96 CD cases some patients showed paraneoplastic pemphigus 1 (1.0%), POEMS syndrome 2 (2.1%), TAFRO syndrome 7 (7.3%), or 2 (2.1%) transformed to lymphoma. The detailed clinical data are analyzed in Table 1.

**Table 1:** Clinical characteristics of 96 patients with Castleman disease

Item	Total	UCD (*n* = 52)	MCD (*n* = 44)	*P *value
Age	46.5 (14–77)	41 (14–77)	53 (24–77)	**0.001**
Gender				
Male	49 (51.0%)	24 (46.2%)	25 (56.8%)	0.298
Female	47 (49.0%)	28 (53.8%)	19 (43.2%)	
Histological subtype				
HV	55 (57.3%)	46 (88.5%)	9 (20.5%)	**0.000**
PC	31 (32.3%)	5 (9.6%)	26 (59.1%)	
Mix	10 (10.4%)	1 (1.9%)	9 (20.5%)	
Leukocyte, 10^9^/L	5.6 (1.2–15.2)	51 (2.8–14.3)	6.5 (1.2–15.2)	0.366
Hemoglobin, g/L	127 (48–174)	137 (86–174)	97 (48–151)	**0.000**
Platelet, 10^9^/L	202 (23–592)	211 (115–444)	169.0 (23–592)	**0.024**
C-reactive protein, mg/L	21.9 (0–335.0)	5.4 (0–159)	42.7 (0–335)	**0.012**
Serum albumin, g/L	41.4 (13.3–59.1)	46.9 (35.3–59.1)	35.7 (13.3–51.2)	**0.000**
Serum globulin, g/L	26.4 (15.6–82.0)	25.0 (18.8–60.2)	30.5 (15.6–82.0)	**0.000**
Serum creatinine, µmol/L	65 (36–245)	60.5 (36–220)	74 (37–245)	0.053
LDH Ul/L	194 (105–475)	211 (128–231)	184 (105–475)	0.498
Fever (≥ 37.5 °C)	16 (16.7%)	1 (1.9%)	15 (34.1%)	
Pleural effusion and/or ascites	18 (18.8%)	1 (1.9%)	17 (38.6%)	
Hepatosplenomegaly	22 (21.9%)	1 (1.9%)	21 (47.7%)	
Thrombocytopenia	14 (14.6%)	0	14 (31.8%)	
Renal dysfunction	12 (12.5%)	0	12 (27.3%)	
Myelofibrosis	5 (5.2%)	0	5 (11.4%)	
PNP	1 (1.0%)	1 (1.9%)	0	
POEMS syndrome	2 (2.1%)	0	2 (4.5%)	
TAFRO syndrome	7 (7.3%)	0	7 (15.9%)	
Progress to lymphoma	2 (2.1%)	0	2 (4.5%)	

Continuous variables were described using median (range) analyzed by Mann–whitney *U* test and categorical variables were described using frequency (percentage) compared by Pearson *χ*^2^ test. Significant *P* values are in bold

*UCD* unicentric Castleman disease, *MCD* multicentric Castleman disease, *HV* hyaline vascular, *PC* plasma cell, *Mix* mixed cellular,* PNP* paraneoplastic pemphigus, *POEMS* polyneuropathy, organomegaly, endocrinopathy, M-protein, and skin abnormalities, *TAFRO* thrombocytopenia, anasarca, myelofibrosis, renal dysfunction, organ enlargement

We also compared the 52(54.2%) UCD and 44(45.8%) MCD in Table 1. UCD patients were much younger than MCD cases, the median age was 41(14–77) years and 53 (24–77) years, respectively (*P* = 0.001). But there was no difference in the gender distribution, with male cases of 46.2% vs. 56.8%, respectively (*P* = 0.298). The major histological type was HV (88.5%) and PC (59.1%), respectively (*P *< 0.001). Compared with UCD, MCD had lower hemoglobin, platelet counts, and serum albumin, but higher serum globulin and C-reactive protein (all, *P* < 0.05).

### Treatments and survival

All UCD cases of our center had surgery for diagnosis and treatment, and 2 patients were treated with 3 months’ interferon after surgery. In all, 3 patients relapsed. One patient with paraneoplastic pemphigus (PNP) was treated with Rituximab for 4 cycles but died because of bronchiolitis obliterans organizing pneumonia (BOOP). The treatment strategies of MCD patients were heterogeneous. Lymph node biopsies were arranged only for pathological diagnoses. 33 (75.0%) cases underwent chemotherapy, 9 (20.5%) received prednisone only, and the remaining 2 (4.5%) cases had a watch and wait strategy. Among them 5 (11.4%) patients were cured, 23 (52.3%) were stable, 8 (18.2%) died, and the other 8(18.2%) had lost. Treatments and outcomes of MCD are summarized in Table 2. After a median follow-up of 41(range 1–138) months, the 5-year overall survival rate for all CD was 89.6%, and the 5-year overall survival rate for UCD and MCD was 98.1% and 77.7%, respectively (*P* = 0.003). The survival of UCD and MCD were compared in Fig. 2c.

**Table 2:** Treatment and outcomes of 44 patients with multicentric Castleman disease (MCD)

Treatment and status	Case (*n*)	Proportion (%)
Treatments		
Wait and watch	2	4.5
Corticosteroid monotherapy	9	20.5
Chemotherapy^a^	33	75.0
Chemotherapy only	15	34.1
Chemotherapy with R	16	36.4
Chemotherapy with L	5	11.4
Chemotherapy with B	4	9.1
Chemotherapy with T	2	4.5
Status		
ANED	5	11.4
AWD	23	52.3
DEAD	8	18.2
LFU	8	18.2

^a^Chemotherapy including: *CHOP* cyclophosphamide, doxorubicin, vincristine, and prednisone, *COP* cyclophosphamide, vincristine, and prednisone, *E-COP* Etoposide, cyclophosphamide, vincristine, and prednisone, *TCD* Thalidomide, cyclophosphamide, and Dexamethasone, *MP* melphala plus prednisone, *R* Rituximab, *L* Lenalidomide, *B* Bortezomib, *T* Tocilizumab. Among them, 6 patients had combination of at least 2 of R, L, B, T; *ANED* alive, no evidence of disease, *AWD* alive with disease, *DEAD* dead, *LFU* lost follow-up

### TAFRO syndrome

The diagnosis of TAFRO syndrome is often delayed. In our groups, the interval between onset and diagnosis was about 12 (1.5–40) weeks. There were 3 men and 4 women with a median age of 53 (35–66) years, 3 patients with PC, 2 with HV, 2 with Mix, all patients were HHV-8 and HIV-negative. At disease onset, 3 out of 7 cases were accompanied by autoimmune diseases, which were rheumatoid arthritis, mixed connective tissue disease and hypothyroidism, respectively. Most MCD exhibits an indolent clinical course, but the 7 TAFRO syndromes in our center manifested with serious and life-threatening symptoms. The main symptoms included thrombocytopenia (7/7), anasarca (7/7), fever (4/7), renal dysfunction (7/7) and organomegaly (6/7). Among the 7 cases, 1 received prednisone monotherapy, 1 received RD (Rituximab, dexamethasone) and the other 5 cases received CHOP (cyclophosphamide, doxorubicin, vincristine, and prednisone) or COP (cyclophosphamide, vincristine, and prednisone) -like therapy as first-line treatment, 2 of the 5 patients combined with Rituximab. Four patients needed hemodialysis or CRRT (continuous renal replacement therapy) because of progressive renal failure. However, one patient failed to have hemodialysis because of low platelet count and rapid disease progression. According to the 2015 diagnostic criteria for TAFRO syndrome, the disease severity was regarded as 3 with ‘slightly severe’ and 4 with ‘severe’ risk. Overall, 3 patients improved by early treatment, 4 patients died from disease progression, only 1 patient (patient No. 4) achieved complete resolution of all the symptoms after the change to lenalidomide based regimen [the detail of this patient was listed in another article as case 3 (Zhou et al. 2017)]. The main clinical findings and outcomes of the 7 patients with TAFRO syndrome are shown in Table 3.

**Table 3:** Clinical characteristics and outcomes of 7 patients with TAFRO

Patient no	Age/gender	Duration (weeks)	Histopathology	PLT (10^9^/L)	Anasarca	Fever	Cr (µmol/L)	Organ enlargement	CRP (mg/L)	Primary treatment	Additional treatment	Best efficacy	Status	OS (months)	Scores^a^	Risk stratification
1	66/F	12	MIX	83 (24)	1	0	171 (249)	0	85	corticosteroid		SD	LFU	1.5	3/2/1/2	Slightly severe
2	50/M	24	HV	47 (16)	1	0	245 (770)	1	136	R-COP^a^6	Hemodialysis	PR	LFU	23.3	1/2/2/3	Slightly severe
3	44/F	40	HV	28 (2)	1	0	131 (373)	1	53.6	E-COP		PD	DEAD	0.7	3/3/1/3	Severe
4	53/M	2	PC	23 (8)	1	1	97 (126)	1	335.4	RD	L-based regimen	CR	ANED	50.8	2/3/3/1	Severe
5	35/F	1.5	PC	42 (3)	1	1	90 (780)	1	31.6	CHOP		PD	DEAD	1.0	3/3/1/3	Severe
6	65/M	1.5	PC	56 (14)	1	1	66 (506)	1	165	COP	hemodialysis	PR	DEAD	3.5	3/2/2/3	Severe
7	54/F	20	MIX	44 (7)	1	1	75 (233)	1	4.8	R-CHOP	CRRT	PD	DEAD	0.6	1/3/0/3	Slightly severe

*PLT* platelet (at diagnosis/the lowest result), *Fever* T > 37.5 °C,* Cr* serum creatinine (at diagnosis/the highest result), *CRP* C-reactive protein, *COP* cyclophosphamide, vincristine, and prednisone, *RD* rituximab, dexamethasone, *CHOP* cyclophosphamide, doxorubicin, vincristine, and prednisone, *R* rituximab, *E* etoposide, *L* lenalidomide, *CRRT* continuous renal replacement therapy, *LFU* lost follow-up,* DEAD* dead, *ANED* alive, no evidence of disease

^a^Scores, anasarca/thrombocytopenia/fever and/or inflammation / renal insufficiency. (1) anasarca: three points maximum, one point for pleural effusion on imaging, one point for ascites on imaging, one point for pitting edema on physical examination. (2) thrombocytopenia: three points maximum, one point for lowest platelet counts < 100,000/μL, two points for lowest platelet counts < 50,000/μL, three points for lowest platelet counts < 10,000/μL. (3) fever and/or inflammation: three points maximum, one point for fever ≥ 37.5 °C but < 38.0 °C or for CRP ≥ 2 mg/dL but < 10 mg/dL, two points for fever ≥ 38.0 °C but < 39.0 °C or for CRP ≥ 10 mg/dL but < 20 mg/dL, three points for fever ≥ 39.0 °C or for CRP ≥ 20 mg/dL. (4) renal insufficiency: three points maximum, one point for GFR < 60 mL/min/1.73 m^2^, two points for GFR < 30 mL/min/1.73 m^2^, three points for GFR < 15 mL/min/1.73 m^2^ or need for hemodialysis. Risk stratification, insufficient for diagnosis (0–2 points), mild (grade 1) (3–4 points), moderate (grade 2) (5–6 points), slightly severe (grade 3) (7–8 points), severe (grade 4) (9–10 points), very severe (grade 5) (11–12 points)

## Discussion

In this study, we analyzed 7 patients with TAFRO syndrome from 96 de novo CDs, and compared the heterogeneity between UCD and MCD in detail. We conclude that UCD may occur in different parts of the body, patients are generally younger and in good condition. Complete resection of the involved lesion can cure these patients, but paraneoplastic pemphigus may be an unfavorable prognostic factor. However, MCD cases had more constitutional symptoms often associated with laboratory abnormalities. Most of them were treated with chemotherapy and the prognosis was significantly worse compared to patients with UCD. For the first time, we discuss 7 Chinese patients with TAFRO syndrome from a single institution. Their prognosis was poor and these patients had more systemic symptoms than classic MCD patients. Most cases received active treatment even with hemodialysis. Lenalidomide based regimen may be a promising new therapy for TAFRO syndrome.

TAFRO syndrome can occur in any age and any race, but it is more common in East Asian populations, especially Japanese and mostly occurs in the middle-aged and elderly (Alhoulaiby et al. 2017; Coutier et al. 2017; Finocchietto et al. 2015; Kubokawa et al. 2014; Liu et al. 2016; Owattanapanich et al. 2018). 7.3% (7/96) of HIV-negative CD and 15.9% (7/44) of MCD patients in our center were diagnosed with TAFRO syndrome, the rate was higher than the reported rate by Oksenhendler et al. (2018) who described that 7% (2/27) of French iMCD consistent with TAFRO syndrome (Oksenhendler et al. (2018), but lower than the results of Owattanapanich et al. (2018) of 18.2% (6/33). In our study, 3 patients were diagnosed within 2 weeks, and most patients’ Eastern Cooperative Oncology Group (ECOG) performance status  > 1, these patients appear to have more acute or subacute onset and in poor condition, which corresponded with the review of Igawa and Sato (2018). The clinical and laboratory characteristics of TAFRO syndrome are significantly different from iMCD-NOS. It is reported that TAFRO syndrome patients were often associated with an elevated VEGF level and a lower IL-6 level, with a normal level of immunoglobulin (Ig), also characteristic with small lymph nodes, obvious thrombocytopenia, pleural effusion and ascites (Nishimura et al. 2019; Srkalovic et al. 2017). All of the 7 cases in our study had thrombocytopenia, anasarca, and renal dysfunction, but none of them had myelofibrosis. The lowest platelet count downed to 2 × 10^9^/L, and the highest serum creatinine reached 780 µmol/L. On histology patients with TAFRO syndrome tend to have a highly vascular lymph node architecture, most cases were Mix, less frequently HV histology. On the contrary, lymph nodes of iMCD-NOS patients often show the pathological features of the typical PC variant CD, including diffuse follicular zone plasma cell proliferation, germinal center protrusion, most were PC type.

To date, the diagnostic criteria for TAFRO syndrome were proposed by Masaki et al. (2016) and Iwaki et al. (2016), respectively. Masaki et al. (2016) also distinguished TAFRO syndrome into 5 risk grades according to systemic edema, platelet count, fever/inflammation, and glomerular filtration rate (GFR). In our study, 3 out of 7 cases were ‘slightly severe’, the other 4 reached the level of ‘severe’ risk. Nara et al. (2017) reported two cases of TAFRO syndrome in Japan, and the disease severity was regarded as “slightly severe” in case 1 and “severe” in case 2. Masaki et al. (2016) evaluated 18 TAFRO syndrome patients in Japan who were classified as having mild (5.5%), moderate (61.1%), slightly severe (22.2%), severe (11.1%), and very severe (0%) risk. This means that the incidence of high-risk TAFRO syndrome patients in China is higher.

In our study, 4 out of 7 patients died and TAFRO syndrome correlated with significantly poorer survival consistent with other reports (Iwaki et al. 2016; Kubokawa et al. 2014; Yu et al. 2017). The optimal treatment for TAFRO syndrome has not been well established. As reported by Iwaki, the clinical course of TAFRO syndrome is more aggressive and characterized by frequent steroid refractoriness requiring additional therapies (Igawa and Sato 2018). Based on the understanding of iMCD, the current treatment strategies for TAFRO syndrome are mainly divided into four categories, including corticosteroid anti-inflammatory therapy, immunosuppressive therapy, chemotherapy, and some novel drugs such as rituximab, anti-IL-6 receptor Ab, anti-angiogenic drugs, and immunomodulator (van Rhee et al. 2018). Corticosteroids are the first-line treatment options, which may be effective at the onset of the disease, but most patients are prone to recurrence during corticosteroid reduction or withdrawal. Patients who are resistant to corticosteroids or have contraindications, rituximab, and anti-IL-6 receptor Ab may be a choice (Fujiwara et al. 2016; Jain et al. 2015). The efficacy of rituximab in HHV-8-positive MCD patients has been well established (Hoffmann et al. 2011), but its effect on long-term survival of iMCD patients may be inferior to anti-IL-6 receptor Abs (Yu et al. 2017). For patients who have a more acute onset and more severe clinical manifestation, like our cases, lymphoma-related chemotherapy may be considered, but the adverse effect after chemotherapy are obvious, and recurrence may occur in some patients. Besides, novel therapeutic approaches such as Bortezomib, recombinant IL-1 receptor antagonist (Anakinra), especially lenalidomide also have achieved good results in selected patients. The use of thalidomide in MCD and TAFRO syndrome has been reported in several cases (Ramasamy et al. 2012; Tatekawa et al. 2015). Lenalidomide as a functional and structural analog of thalidomide, has greater potency but less peripheral neurotoxicity. Lenalidomide has been shown with anti-inflammatory, anti-angiogenic and immunomodulatory effects. This specific immunomodulation may be effective in malignant plasma diseases, inhibits production of interleukin-6, which is the key cytokine in the pathogenesis of CD. To date, 7 articles were obtained from PUBMED literature search using keyword Lenalidomide and Castleman disease (Adam et al. 2012, 2016; Cai et al. 2019; Szturz et al. 2012, 2013a, b; Zhou et al. 2017). Including one in which we summarized our experience with lenalidomide containing regimen as salvage therapy in 3 relapsed/refractory MCD (Zhou et al. 2017). From those data, we observed an excellent effect of lenalidomide in MCD. Since TAFRO is a subtype of MCD and strongly related to immunity, it is possible that lenalidomide can be a new treatment option for TAFRO syndrome.

Castleman disease, especially TAFRO syndrome, is still a huge challenge for clinicians. At present, researchers still have insufficient understanding of TAFRO syndrome, and the analysis of patients with CD, especially TAFRO syndrome will deepen the understanding of the disease. For CD patients with renal dysfunction, thrombocytopenia, and multiple serous effusions, the possibility of TAFRO syndrome should be considered.
